# An Extreme Clinical Diagnosis: Primary Metastatic Breast Cancer with Complete Bilateral Breast Contour Elimination and Ulceration

**DOI:** 10.3390/diagnostics16111744

**Published:** 2026-06-05

**Authors:** Menelaos Zafrakas, Theodoros Argyriou, Panayiota Papasozomenou, Christos Emmanouilides

**Affiliations:** 1 School of Health Science, International Hellenic University, 57400 Thessaloniki, Greece; 2 European Interbalkan Medical Center, 55535 Thessaloniki, Greece

**Keywords:** primary metastatic breast cancer, ulcerative breast cancer, primary chemotherapy, systemic therapy

## Abstract

A 51-year-old woman was admitted with a malodorous ulceration covering the whole area of both breasts, without visible breast contour or remnants of breast tissue. After excision of a skin nodule an invasive ductal carcinoma was diagnosed; grade-2, hormone receptor (HR)-positive, HER2-negative, Ki-67 at 25%. Computed tomography of the thorax and abdomen showed pulmonary and osseous metastases. Six cycles of systemic chemotherapy with epirubicin and cyclophosphamide at three-week intervals were administered, followed by endocrine therapy with letrozole. Almost four years later, palbociclib became available and it was added to the patient’s treatment. Loco-regional and distant disease control was achieved attaining maximum response at 11 months after initial diagnosis and since then the patient remains progression-free with good quality of life for more than eight years. This is to the best of our knowledge an extreme case of primary metastatic ulcerative breast cancer with complete local tissue destruction and markedly prolonged progression-free survival. As this is a single-case clinical observation, any conclusions have limited generalizability. Given the rarity of primary metastatic ulcerative breast cancer there are no specific evidence-based treatment guidelines available and published studies have high heterogeneity and low level of evidence, necessitating multidisciplinary approach on a case-by-case basis.

**Figure 1:**
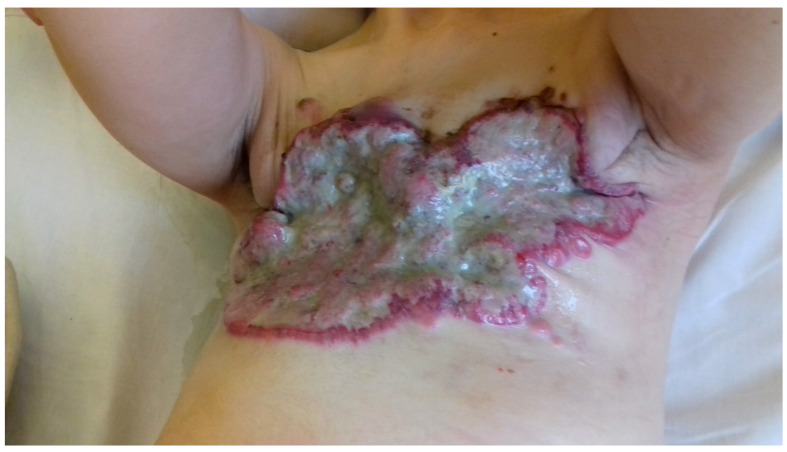
This is to the best of our knowledge an extreme case of primary metastatic ulcerative breast cancer with complete local tissue destruction and markedly prolonged progression-free survival. In January 2018, a 51-year-old woman was admitted with a malodorous ulceration, which covered the whole area of the breasts; both breasts seemed to be disintegrated, the breast contours were completely eliminated and there were no visible remnants of breast tissue. According to her relatives who accompanied her, the patient was hiding her ailment for an undetermined period of time and consented to receive medical care only after increasing foul odor could no longer be concealed. The patient conceded that she should have sought medical care earlier and complained that although she had recently visited two different medical care facilities appropriate care was not offered as she was deemed terminal. Our multi-disciplinary team opted for further treatment after a careful step-wise diagnostic and therapeutic approach. First a skin nodule was excised under local anesthesia. Histological and immunohistochemical examination showed an invasive ductal carcinoma, grade-2, estrogen receptor (ER)-positive, progesterone receptor (PR)-positive, HER2-negative and Ki-67 expression at 25%. In parallel, computed tomography (CT) of the thorax and abdomen showed pulmonary and osseous metastases. Based on evidence from reports that were available at the time [1,2] suggesting that primary chemotherapy may benefit patients with ulcerative breast cancer, six cycles of systemic chemotherapy with epirubicin and cyclophosphamide at three-week intervals were administered until July 2018, achieving a partial response. Subsequently, endocrine therapy with daily administration of letrozole was initiated.

**Figure 2:**
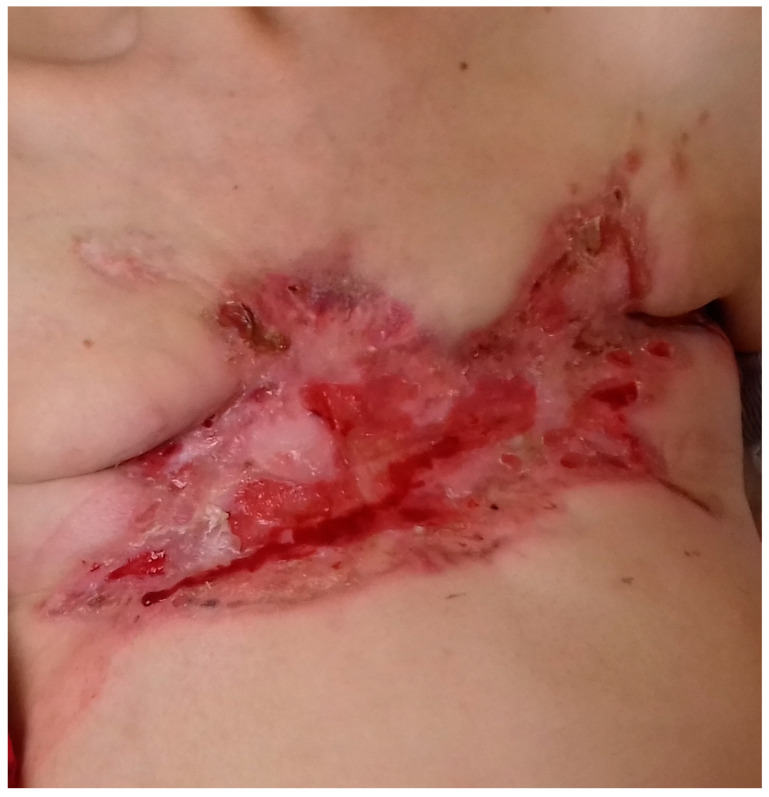
The ulcer covering the area of both breasts diminished markedly in size after systemic therapy and regular local care with local antiseptic solutions and sterile gauze dressings; surgical debridement was not feasible due to the extent of disease. Maximum control of loco-regional and distant disease was achieved in November 2018, i.e., 11 months after initial diagnosis, necessitating only local care of the remaining ulcerative lesions; endocrine therapy with letrozole was continued. In parallel, zoledronic acid was administered, initially with 12 monthly infusions and then three-monthly thereafter. In December 2021 palbociclib became available and its daily administration was added to the patient’s treatment. According to current treatment guidelines for hormone receptor (HR)-positive, HER2-negative metastatic breast cancer [3,4,5,6,7], today palbociclib would have been administered upfront together with letrozole, and it would be debatable if primary chemotherapy should have been given prior to endocrine therapy.

**Figure 3:**
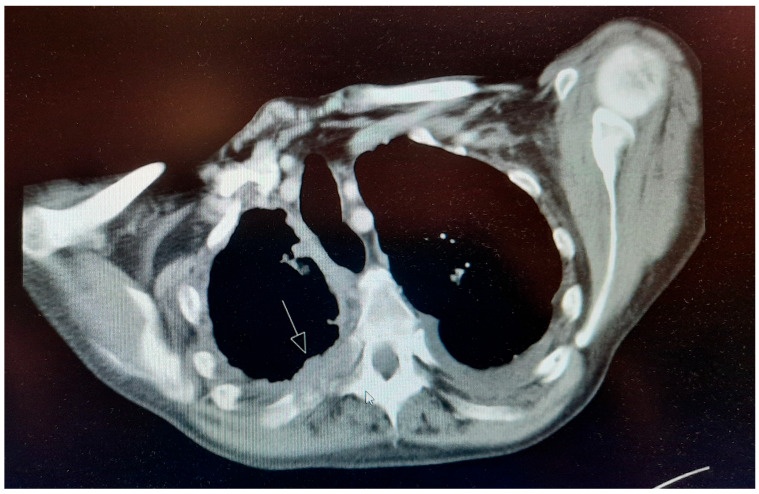
Maximum control of loco-regional and distant disease was achieved in November 2018, i.e., 11 months after initial diagnosis; on CT, only a small pleural effusion (arrow) was visible. The patient has been monitored since then with serial clinical and imaging examinations with CT of the thorax and abdomen, and measurements of tumor marker levels in peripheral blood (CA-15.3, CEA and CA.125), which remained within the normal range; the patient remains progression-free with good quality of life since then, more than eight years after initial diagnosis. This extreme case highlights the necessity of increasing breast cancer awareness among women and awareness of breast cancer symptoms and underscores the impact of popular notions preventing early diagnosis [8]. This study has the inherent limitation of restricted generalizability of conclusions derived from single-case clinical observations. Due to the rarity of ulcerative breast cancer existing evidence is scarce and there are no clear guidelines for treatment available. A recent systematic review on therapeutic strategies for fungating and ulcerating breast cancers [9] showed that published studies have high heterogeneity and low level of evidence. In any case, local care of ulcerative breast cancer lesions is important in order to prevent microbial contaminations [10] and bleeding, as cases of excessive bleeding have been reported, necessitating treatment with local hemostatic agents [11] or radiation therapy [12,13]. In selected cases, sophisticated reconstructive procedures have been reported [14,15]. Previous reports suggest that primary chemotherapy seems to benefit patients with inoperable, ulcerative breast cancer [1,2]. However, individualized treatment should be decided by a multidisciplinary team on a case-by-case basis, based on disease stage, and tumor molecular phenotype according to current clinical guidelines [3,4,5,6].

## Data Availability

Further information regarding the case is available on reasonable request from the corresponding author.

## References

[1] Fiegl M., Kaufmann H., Steger G.G. (2001). Ulcerative breast cancer: Case report and review of management. Breast J..

[2] Kakagia D., Trichas M., Papadopoulos N., Tsalkidis A., Jivannakis T., Tamiolakis D. (2004). Ulcerative locally advanced breast cancer: The efficacy of combined anthracycline-based and hormonal therapy. Eur. J. Gynaecol. Oncol..

[3] NCCN Clinical Practice Guidelines in Oncology: Breast Cancer (Version [3.2026]).

[4] Gennari A., André F., Barrios C.H., Cortés J., de Azambuja E., DeMichele A., Dent R., Fenlon D., Gligorov J., Hurvitz S.A. (2021). ESMO Clinical Practice Guideline for the diagnosis, staging and treatment of patients with metastatic breast cancer. Ann. Oncol..

[5] Thill M., Janni W., Albert U.S., Banys-Paluchowski M., Bartsch R., Bauerfeind I., Bjelic-Radisic V., Blohmer J., Budach W., Dall P. (2025). AGO Recommendations for the Diagnosis and Treatment of Patients with Locally Advanced and Metastatic Breast Cancer: Update 2025. Breast Care.

[6] Ditsch N., Untch M., Fasching P.A., Briest S., Ettl J., Haidinger R., Lüftner D., Maurer C., Müller V., Park-Simon T.W. (2026). ABC8 Consensus: Assessment by a German Group of Experts. Breast Care.

[7] Finn R.S., Martin M., Rugo H.S., Jones S., Im S.A., Gelmon K., Harbeck N., Lipatov O.N., Walshe J.M., Moulder S. (2016). Palbociclib and Letrozole in Advanced Breast Cancer. N. Engl. J. Med..

[8] Fejer A., Atbaei M.A., Zand A., Varjas T., Zsuzsanna Kiss Z. (2026). Psychometric Properties of the Breast Cancer Awareness Measure (Breast-CAM): A Systematic Review and Meta-Analysis. Cancers.

[9] Zagardo V., Harikar M., Ferini G. (2025). Therapeutic strategies for fungating and ulcerating breast cancers: A systematic review and narrative synthesis. Breast.

[10] Mourad A., Lorenz N., Patel K., Patel I., Pattathan M. (2026). Fungating Breast Carcinoma Complicated by Carbapenem-Resistant Pseudomonas aeruginosa Infection in a Patient With Severe Blood-Injection-Injury Phobia: A Case Report. Cureus.

[11] Abid H., Soliman M., Williams K. (2024). The Use of Floseal Hemostatic Agent for Local Bleeding Control in Fungating Breast Cancer: A Case Report and Review of Literature. Cureus.

[12] Kokubo Y., Ashida R., Tokuda P.J.K., Mitsuyoshi T., Imagumbai T., Kokubo M. (2025). A case of long-term relief from bleeding due to fungating breast cancer in an elderly patient after 8-Gy single-fraction radiation therapy. Int. Cancer Conf. J..

[13] Kil W.J., Smith W., Muchnik E., Collins R., Cousins D. (2025). Adaptive Repeat Quad Shot Radiation Therapy for Uncontrolled Symptomatic Fungating or Skin-Infiltrating Primary and Regional Nodes in Patients with Metastatic Breast Cancer: Durable In-Field Tumor Control Without Interrupting Systemic Therapy. Adv. Radiat. Oncol..

[14] Abdallah A., Abdelwahab K., Awny S., Zuhdy M., Hamdy O., Atallah K., Elfeky A., Hegazy M.A.F., Metwally I.H. (2023). Fungating and Ulcerating Breast Cancer: Wound Closure Algorithm, Complications, and Survival Trends. Indian J. Surg. Oncol..

[15] Sood A., Daniali L.N., Rezzadeh K.S., Lee E.S., Keith J. (2015). Management and Reconstruction in the Breast Cancer Patient With a Fungating T4b Tumor. Eplasty.

